# P-2001. Limited Yield of Weekly SARS-CoV-2 Surveillance Testing Among Asymptomatic Hematopoietic Cell Transplant and Chimeric Antigen Receptor T-Cell Therapy Recipients

**DOI:** 10.1093/ofid/ofae631.2158

**Published:** 2025-01-29

**Authors:** Marie H Wilson, Elizabeth M Krantz, Steven A Pergam, Marco Mielcarek, Suni Elgar, Emily A Rosen, Michelle Swetky, Salma Walji, Catherine Liu, Seth Cohen, Denise McCulloch

**Affiliations:** Fred Hutchinson Cancer Center, Seattle, Washington; Fred Hutch Cancer Center, Seattle, Washington; Fred Hutchinson Cancer Center; University of Washington, Seattle, WA; Fred Hutchinson Cancer Research Center; University of Washington, Seattle, Washington; Fred Hutchinson Cancer Center, Seattle, Washington; Fred Hutchinson Cancer Center / University of Washington, Seattle, Washington; Fred Hutchinson Cancer Center, Seattle, Washington; Fred Hutchinson Cancer Center, Seattle, Washington; Fred Hutchinson Cancer Center, Seattle, Washington; University of Washington, Seattle, Washington; Fred Hutchinson Cancer Center, Seattle, Washington

## Abstract

**Background:**

Surveillance testing for severe acute respiratory syndrome coronavirus 2 (SARS-CoV-2) among asymptomatic patients is used in some healthcare settings to prevent outbreaks. We examined the utility of weekly SARS-CoV-2 testing among asymptomatic hematopoietic cell transplant (HCT) and chimeric antigen receptor T-cell (CART) therapy recipients at a comprehensive cancer center.

**Figure:**
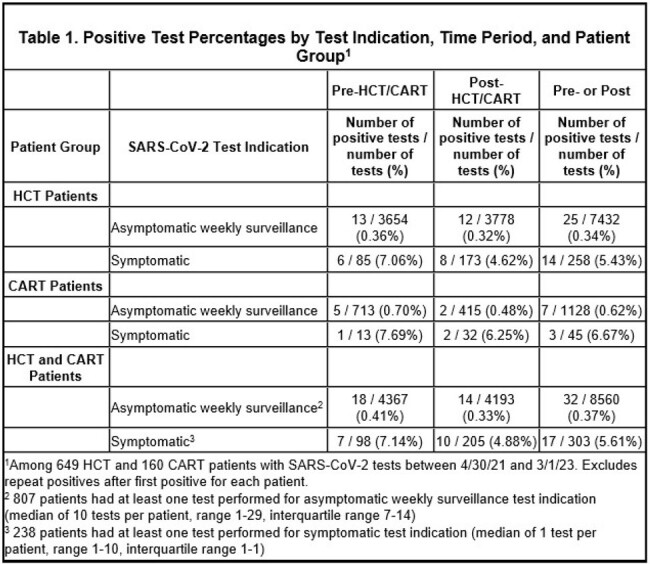

**Methods:**

We retrieved nasopharyngeal and nasal SARS-CoV-2 polymerase chain reaction (PCR) results and test indications from a cohort of HCT and CART recipients from April 30, 2021 to March 1, 2023, when weekly asymptomatic surveillance was performed. Test results were captured between patients’ Center arrival dates and either discharge home, day before a second HCT or CART infusion, death, date of last contact, or March 1, 2023. Only the first positive test per patient was included in the analysis; positive test results prompted chart review to determine false positives, symptom development, and impact on cancer care.

**Figure 1.**
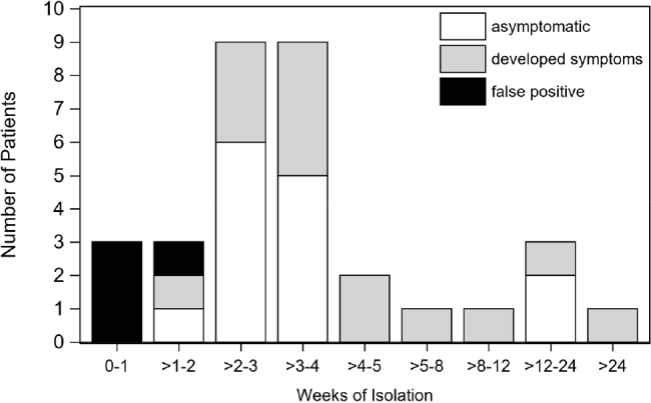
Weeks of isolation among 32 patients testing positive for SARS-CoV-2 from asymptomatic weekly surveillance testing.

Empty bars represent patients with asymptomatic infection (n=14), light gray bars represent those who developed symptoms (n=14), and black bars represent false positives (n=4).

**Results:**

8863 PCR tests were obtained from 809 patients, including 8560 weekly surveillance tests from 807 asymptomatic patients (median of 10 tests per patient, range 1-29), and 303 tests from 238 symptomatic patients. Tests performed among asymptomatic patients were less frequently positive (0.4%) than those performed among symptomatic patients (5.6%; Table 1). Of the 32/807 (4%) patients testing positive by asymptomatic weekly surveillance, 14 (44%) developed symptoms consistent with Coronavirus Disease 2019 (COVID-19), 14 (44%) were asymptomatic, and 4 (13%) were false positives. The 32 positive patients were isolated for a median of 22 days (range 0-266) (Fig 1). 13 (41%) patients with true positives and 1 (3%) patient with a false-positive had delays in cancer care (Fig 2).

**Figure 2.**
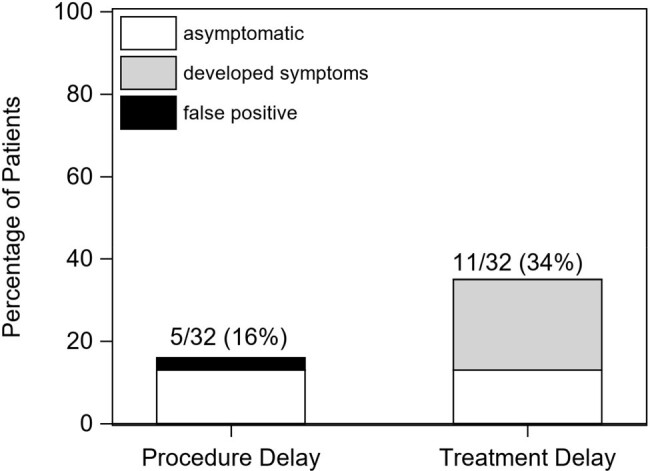
Percentage of patients with delays in procedures or treatment, among 32 patients testing positive for SARS-CoV-2 from asymptomatic weekly surveillance testing.

Includes 19 patients testing positive pre-HCT/CART and 17 testing positive post-HCT/CART. Procedures were defined as medical interventions required prior to HCT or CART (e.g., port placement, pulmonary function tests). Treatment was defined as therapeutic agents/modalities associated with cancer care, including HCT or CART. Empty bars represent patients with asymptomatic infection (4 with procedure delay, 4 with treatment delay), light gray bars represent those who developed symptoms (0 with procedure delay, 7 with treatment delay), and black bars represent false positives (1 with procedure delay, 0 with treatment delay). Two patients had delays in both procedures and treatment; 14/32 (44%) had delays in either procedures or treatment.

**Conclusion:**

Weekly SARS-CoV-2 surveillance testing among asymptomatic yet high-risk HCT and CART patients at a comprehensive cancer center over a two-year period identified 28 patients (3% of those tested) with pre-symptomatic or asymptomatic infection. Considering costs of screening, and effectiveness of infection prevention strategies, including symptom screening, universal masking, and access to rapid symptomatic testing, our data suggest limited value of weekly asymptomatic screening in this setting.

**Disclosures:**

Steven A. Pergam, MD, MPH, Cidara: Advisor/Consultant|F2G: Advisor/Consultant|Global Life Technologies: Grant/Research Support|Symbio: Advisor/Consultant Catherine Liu, MD, Pfizer: Grant/Research Support

